# Bioinformatic analysis reveals the expression of unique transcriptomic signatures in Zika virus infected human neural stem cells

**DOI:** 10.1186/s13578-016-0110-x

**Published:** 2016-06-10

**Authors:** Alyssa J. Rolfe, Dale B. Bosco, Jingying Wang, Richard S. Nowakowski, Jianqing Fan, Yi Ren

**Affiliations:** Department of Biomedical Sciences, College of Medicine, Florida State University, 1115 West Street, Tallahassee, FL 32306 USA; Statistical Laboratory, Princeton University, Princeton, NJ 08540 USA

## Abstract

**Background:**

The single-stranded RNA *Flavivirus*, Zika virus (ZIKV), has recently re-emerged and spread rapidly across the western hemisphere’s equatorial countries, primarily through *Aedes* mosquito transmission. While symptoms in adult infections appear to be self-limiting and mild, severe birth defects, such as microcephaly, have been linked to infection during early pregnancy. Recently, Tang et al. (Cell Stem Cell 2016, doi: 10.1016/j.stem.2016.02.016) demonstrated that ZIKV efficiently infects induced pluripotent stem cell (iPSC) derived human neural progenitor cells (hNPCs), resulting in cell cycle abnormalities and apoptosis. Consequently, hNPCs are a suggested ZIKV target.

**Methods:**

We analyzed the transcriptomic sequencing (RNA-seq) data (GEO: GSE78711) of ZIKV (Strain: MR766) infected hNPCs. For comparison to the ZIKV-infected hNPCs, the expression data from hNPCs infected with human cytomegalovirus (CMV) (Strain: AD169) was used (GEO: GSE35295). Utilizing a combination of Gene Ontology, database of human diseases, and pathway analysis, we generated a putative systemic model of infection supported by known molecular pathways of other highly related viruses.

**Results:**

We analyzed RNA-sequencing data for transcript expression alterations in ZIKV-infected hNPCs, and then compared them to expression patterns of iPSC-derived hNPCs infected with CMV, a virus that can also induce severe congenital neurological defects in developing fetuses. We demonstrate for the first time that many of cellular pathways correlate with clinical pathologies following ZIKV infection such as microcephaly, congenital nervous system disorders and epilepsy. Furthermore, ZIKV activates several inflammatory signals within infected hNPCs that are implicated in innate and acquired immune responses, while CMV-infected hNPCs showed limited representation of these pathways. Moreover, several genes related to pathogen responses are significantly upregulated upon ZIKV infection, but not perturbed in CMV-infected hNPCs.

**Conclusion:**

The presented study is the first to report enrichment of numerous pro-inflammatory pathways in ZIKV-infected hNPCs, indicating that hNPCs are capable of signaling through canonical pro-inflammatory pathways following viral infection. By defining gene expression profiles, new factors in the pathogenesis of ZIKV were identified which could help develop new therapeutic strategies.

## Background

Zika Virus (ZIKV) is a single-stranded positive sense RNA *Flavivirus* [1] that is primarily transmitted though the *Aedes* mosquito [2]. Until recently this virus had remained relatively obscure, with the exception of a few scattered outbreaks [1–3]. Currently, 26 countries across South and Central America have reported widespread ZIKV infections, with cases also emerging in Europe and the United States [4]. Typically, adult ZIKV infections are mild or asymptomatic, with exanthema, fever, conjunctivitis, and joint pain being the most common symptoms [2, 5]. However, ZIKV infection is also believed to be related to the concurrent increase in adult Guillain–Barré syndrome (GBS) cases [4]. This is supported by a case controlled prospective study of the French Polynesian outbreak, where ZIKV was linked with increased incidence of GBS [3]. Aside from its effects in adults, ZIKV infection of pregnant women can result in birth defects ranging from low birth weight to craniofacial and eye abnormalities [6], as well as microcephaly [7, 8]. The detection of ZIKV in fetal brain tissues indicates that ZIKV can also cross the placental barrier [9]. Consequently, curbing the current ZIKV epidemic has become a top priority for a number of international health initiatives.

Prompt laboratory-based efforts by Tang et al. provided RNA-seq data (GEO: GSE78711) from ZIKV-infected human neural progenitor cells (hNPCs). They found that when compared to neurons and embryonic stem cells, ZIKV infects hNPCs with high efficiency, and that infection results in abnormal cell cycle dynamics and apoptosis [9]. For the current study, we analyzed this dataset using bioinformatic methods to uncover links between neuro-inflammatory pathways and ZIKV infection of hNPCs, as well as its correlation with clinical neurological symptoms. Since congenital CMV infection is also associated with higher incidence of neurological birth defects [10], a CMV-infected hNPC dataset (GEO: GSE19345) was used for comparison [11]. We demonstrate for the first time that ZIKV activates several inflammatory signals within infected hNPCs that are implicated in both innate and acquired immune responses. Moreover, several genes related to pathogen responses are significantly up-regulated in response to ZIKV infection, but not perturbed in CMV-infected hNPCs. Our approach offers novel insight into the potential mechanisms underlying ZIKV infection and provides useful directions for future clinical research.

## Results

### Comprehensive gene set gene ontology analysis

**Fig. 1:**
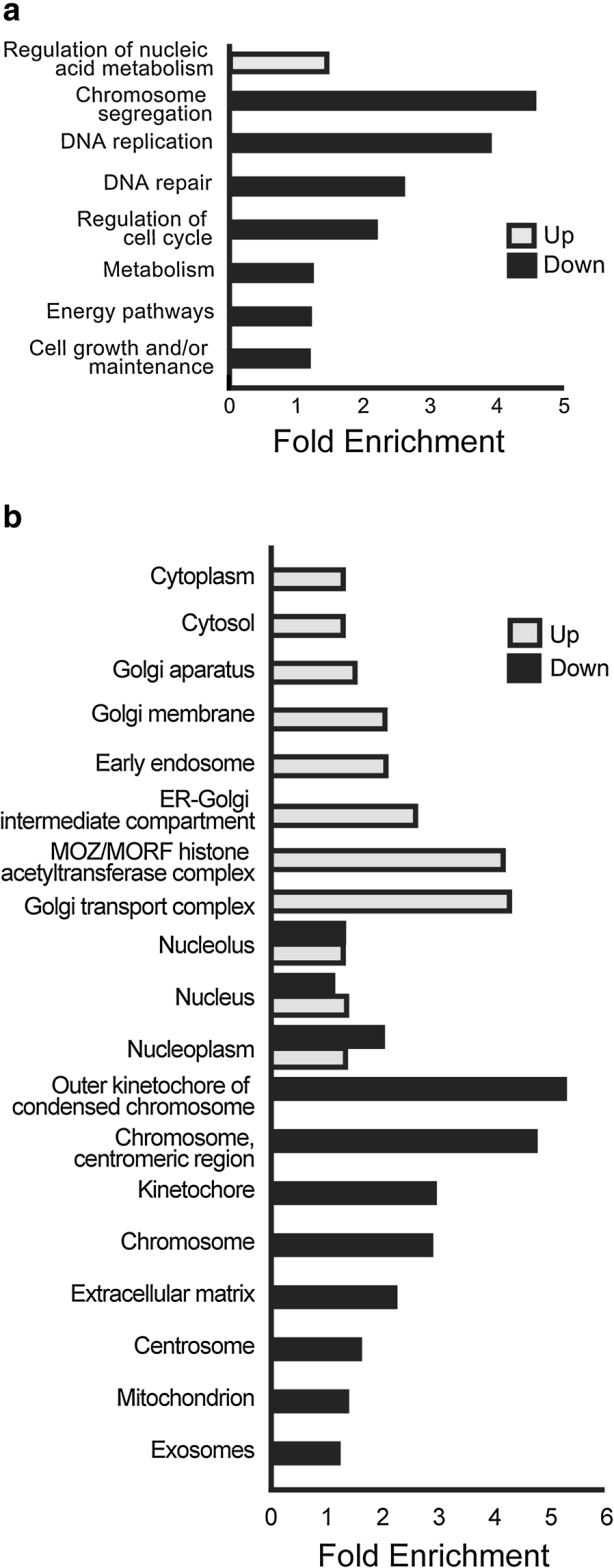
Comprehensive gene set gene ontology analysis. Gene ontology analysis was performed on hNPC genes whose expression was significantly altered following infection with ZIKV for 56 h. Transcript enrichment significance was determined using the human protein coding genome as the reference background. **a** Regulation of nucleic acid metabolism was the only biological process sub-ontology enriched with ZIKV up-regulated genes. Down-regulated genes were enriched for pathways related to cell cycle regulation and general metabolism. All significant terms are presented. **b** Cellular component enrichment indicated down-regulated genes generally associated with chromosomal regions, the extracellular matrix, and centrosomes. Up-regulated genes showed the most enrichment for the Golgi apparatus. Both up- and down-regulated genes were significantly enriched in nuclear sites. Enrichment scores were sorted by the most significant terms, and the top 11 compartments are represented. (q < 0.05)

Comparison of the mock-infected samples to the ZIKV-infected samples using a modified *t*-test yields a total of 3443 genes up-regulated and 3421 down-regulated. The top 30 highest absolute fold-change (FC) genes are reported in Tables 1 and 2 along with their associated Entrez IDs and p-values. Gene Ontology (GO) term analysis showed that the up-regulated gene set was exclusively enriched in processes related to nucleic acid metabolism regulation (Fig. 1a). Since replication of the viral genome requires reallocation of host nucleotides [12], this finding was unsurprising. Also, the down-regulated gene set was enriched for DNA metabolism processes, although with emphasis on cell cycle regulation. These findings were consistent with evidence showing ZIKV infection results in the down-regulation of cell cycle genes, impaired cell cycle progression through G_1_ [9], and decreased proliferation [13] in vitro. Of the ZIKV down-regulated genes, the biological process most enriched proved to be chromosome segregation. This finding provides an interesting link to known causes of hereditary microcephaly, as abnormal brain development and reduced volume often are the result of dysregulation of chromosome segregation [14, 15].

**Table 1:** Top 30 genes up-regulated in ZIKV infected hNPCs compared to controls

Gene symbol	Entrez	Gene name	Log2 FC	p value
IFIT2	3433	Interferon-induced protein with tetratricopeptide repeats 2	5.22967	0.0051
TNFRSF14	8764	Tumor necrosis factor receptor superfamily, member 14	3.4467	0.00005
BHLHE41	79365	Basic helix-loop-helix family, member e41	3.41193	0.00005
SNORD116-4	100033416	Small nucleolar RNA, C/D box 116-4	3.3541	0.00065
CEBPB	1051	CCAAT/enhancer binding protein (C/EBP), beta	3.08608	0.00005
SEC24D	9871	SEC24 family, member D (*S*. *cerevisiae*)	3.06648	0.00005
CHAC1	79094	ChaC, cation transport regulator homolog 1 (*E. coli*)	2.98802	0.00005
CREB3L1	90993	cAMP responsive element binding protein 3-like 1	2.9718	0.00005
DDIT3	1649	DNA-damage-inducible transcript 3	2.94172	0.00005
STC2	8614	Stanniocalcin 2	2.85446	0.00005
IL20RB	53833	Interleukin 20 receptor beta	2.74302	0.00005
ADM2	79924	Adrenomedullin 2	2.73791	0.00005
DDR2	4921	Discoidin domain receptor tyrosine kinase 2	2.68283	0.00005
ULBP1	80329	UL16 binding protein 1	2.6589	0.00005
SLC7A11	23657	Solute carrier family 7 (anionic amino acid transporter light chain, xc-system), member 11	2.55855	0.00005
GABRR2	2570	Gamma-aminobutyric acid (GABA) A receptor, rho 2	2.54697	0.00025
FOSL1	8061	FOS-like antigen 1	2.54626	0.00005
RELB	5971	v-rel reticuloendotheliosis viral oncogene homolog B	2.52167	0.00005
BHLHE40	8553	Basic helix-loop-helix family, member e40	2.50278	0.00005
INHBE	83729	Inhibin, beta E	2.48416	0.00005
XBP1	7494	X-box binding protein 1	2.48392	0.00005
SELPLG	6404	Selectin P ligand	2.47628	0.00005
SLC7A5	8140	Solute carrier family 7 (amino acid transporter light chain, L system), member 5	2.44283	0.00005
ARHGAP9	64333	Rho GTPase activating protein 9	2.42899	0.00005
HERPUD1	9709	Homocysteine-inducible, endoplasmic reticulum stress-inducible, ubiquitin-like domain member 1	2.38525	0.00005
KLF15	28999	Kruppel-like factor 15	2.36633	0.00005
CDC42EP1	11135	CDC42 effector protein (Rho GTPase binding) 1	2.35073	0.00005
CEBPD	1052	CCAAT/enhancer binding protein (C/EBP), delta	2.34979	0.00025
GAL3ST2	64090	Galactose-3-*O*-sulfotransferase 2	2.31589	0.0021
FAM129A	116496	Family with sequence similarity 129, member A	2.28808	0.00005

**Table 2:** Top 30 genes down-regulated in ZIKV infected hNPCs compared to controls

Gene symbol	Entrez	Gene name	Log2 FC	p-value
SIGLEC10	89790	Sialic acid binding Ig-like lectin 10	−3.60664	0.00005
CDC20B	166979	Cell division cycle 20 homolog B (*S*. *cerevisiae*)	−3.44083	0.0002
OR51E2	81285	Olfactory receptor, family 51, subfamily E, member 2	−3.05742	0.0001
CCNO	10309	Cyclin O	−3.00562	0.00005
SLCO4A1	28231	Solute carrier organic anion transporter family, member 4A1	−2.97508	0.0229
SGPP2	130367	Sphingosine-1-phosphate phosphatase 2	−2.69025	0.00035
RRM2	6241	Ribonucleotide reductase M2	−2.63783	0.00005
SFXN2	118980	Sideroflexin 2	−2.61672	0.00005
COLEC12	81035	Collectin sub-family member 12	−2.60458	0.00005
CHRNA1	1134	Cholinergic receptor, nicotinic, alpha 1 (muscle)	−2.5688	0.00005
PRR22	163154	Proline rich 22	−2.54683	0.00045
DHFR	1719	Dihydrofolate reductase	−2.53145	0.00005
MFI2	4241	Antigen p97 (melanoma associated) identified by monoclonal antibodies 133.2 and 96.5	−2.50748	0.00005
MAPK15	225689	Mitogen-activated protein kinase 15	−2.46203	0.00005
C21orf58	54058	Chromosome 21 open reading frame 58	−2.39697	0.00005
SPEF1	25876	Sperm flagellar 1	−2.39565	0.00005
GHRL	51738	Ghrelin/obestatin prepropeptide	−2.35804	0.00805
ATG16L2	89849	Autophagy related 16-like 2 (*S*. *cerevisiae*)	−2.34225	0.00005
CXCL14	9547	Chemokine (C-X-C motif) ligand 14	−2.33189	0.0015
SUSD2	56241	Sushi domain containing 2	−2.28898	0.00005
C6orf118	168090	Chromosome 6 open reading frame 118	−2.27987	0.00005
DLX6-AS1	285987	DLX6 antisense RNA 1	−2.26662	0.00005
ADAM28	10863	ADAM metallopeptidase domain 28	−2.25906	0.00005
NXPH2	11249	Neurexophilin 2	−2.22856	0.00005
AQP7P1	375719	Aquaporin 7 pseudogene 1	−2.2255	0.00005
EFCAB1	79645	EF-hand calcium binding domain 1	−2.19918	0.00005
WNT8B	7479	Wingless-type MMTV integration site family, member 8B	−2.19366	0.00005
PI15	51050	Peptidase inhibitor 15	−2.17615	0.00005
C1orf189	388701	Chromosome 1 open reading frame 189	−2.17531	0.01255
OLFML3	56944	Olfactomedin-like 3	−2.16835	0.0016

By also assessing cellular compartment sub-ontology, we elucidated the cellular location of the infection induced gene expression alterations (Fig. 1b). Genes down-regulated by ZIKV were highly enriched in centromeric and nuclear sites, the extracellular matrix, and mitochondria. Furthermore, down-regulated genes were associated with exosomes, suggesting that the infection can influence exosome biology. Up-regulated genes were enriched specifically in the Golgi complex, the cytoplasm, and the monocytic leukemia zinc-finger protein/related factor (MOZ/MORF) histone acetyltransferase complex, which serves as a regulator of neural and hematopoietic stem-cell identity [16].

### ZIKV DEGs represented in canonical human neurological diseases

**Fig. 2:**
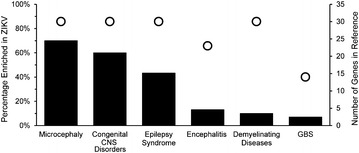
ZIKV DEGs mapped to canonical human disease gene sets. Using the MalaCards database of human diseases, diseases with established clinical significance to ZIKV infection were queried. ZIKV DEGs were then compared to reference gene list for each disease to determine a percentage of representation. Diseases related to congenital CNS disorders were most highly represented with ZIKV DEGs. Although less, representation of adult inflammatory and demyelinating diseases were also observed. (*Bars* % of ZIKV induced genes present in the reference list) (*Open circles* total number of genes in the disease reference list)

To investigate whether ZIKV induced differentially expressed genes (DEGs) are related to the observed clinical phenotypes, comparisons were made between significant ZIKV DEGs and those related to the pathogenesis of human neurological diseases (Fig. 2) (Table 3). Considering that hNPCs were used for the RNA-seq study, our inquiry was limited to diseases of the nervous system. Using the MalaCards [17] integrated searchable database of human diseases, we selected six conditions for comparison. Each condition is a parent disease relating sub-categories of more specific diseases. For each condition, the number of ZIKV DEGs present in the MalaCards reference list is reported as the represented percentage. The number of total genes in each MalaCards reference list is plotted along the second y-axis (scatterplot). The condition with the most representation proved to be microcephaly, with 70 % of the 30 reference list genes present in the ZIKV DEGs. Since, epilepsy is a common microcephaly co-morbidly [18], it was unsurprising to discover there was 43 % representation of related genes. The more general group of congenital nervous system disorders also displayed a similarly high representation of ZIKV DEGs at 60 % of the total reference list. Since, there has also been an increase in reported cases of adult GBS in ZIKV affected areas [2, 3], we investigated encephalitis, demyelinating diseases, and GBS specifically. While representation of ZIKV DEGs in these disease classifications were present, it was significantly less than congenital central nervous system (CNS) diseases.

**Table 3:** Neurological clinical phenotypes associated with ZIKV DEGs

Disease	Gene symbol	Gene name
Microcephaly	DYRK1A	Dual-specificity tyrosine-(Y)-phosphorylation regulated kinase 1A
	MCPH1	Microcephalin 1
	CASK	Calcium/calmodulin-dependent serine protein kinase (MAGUK family)
	PHGDH	Phosphoglycerate dehydrogenase
	ARFGEF2	ADP-ribosylation factor guanine nucleotide-exchange factor 2
	ASPM	Asp (abnormal spindle) homolog, microcephaly associated
	CENPJ	Centromere protein J
	CEP152	Centrosomal protein 152 kDa
	SLC25A19	Solute carrier family 25 (mitochondrial thiamine pyrophosphate carrier), member 19
	WDR62	WD repeat domain 62
	CDK5RAP2	CDK5 regulatory subunit associated protein 2
	CASC5	Cancer susceptibility candidate 5
	NBN	Nibrin
	EFTUD2	Elongation factor Tu GTP binding domain containing 2
	IER3IP1	Immediate early response 3 interacting protein 1
	STIL	SCL/TAL1 interrupting locus
	CEP135	Centrosomal protein 135 kDa
	ZNF335	Zinc finger protein 335
	NDE1	nudE nuclear distribution E homolog 1
	DIAPH1	Diaphanous homolog 1
	KIF11	Kinesin family member 11
Congenital nervous system disorders	MCPH1	Microcephalin 1
	ARFGEF2	ADP-ribosylation factor guanine nucleotide-exchange factor 2 (brefeldin A-inhibited)
	TGIF1	TGFB-induced factor homeobox 1
	GRIN2B	Glutamate receptor, ionotropic, *N*-methyl d-aspartate 2B
	SLC12A5	Solute carrier family 12 (potassium/chloride transporter), member 5
	FLNA	Filamin A, alpha
	SIX3	SIX homeobox 3
	SHH	Sonic hedgehog
	CDK5RAP2	CDK5 regulatory subunit associated protein 2
	NDE1	nudE nuclear distribution E homolog 1
	WDR62	WD repeat domain 62
	ASPM	Asp (abnormal spindle) homolog, microcephaly associated
	CENPJ	Centromere protein J
	STIL	SCL/TAL1 interrupting locus
	FKTN	Fukutin
	POMGNT1	Protein O−linked mannose beta1,2−N−acetylglucosaminyltransferase
	CEP152	Centrosomal protein 152 kDa
	POMT2	Protein−O−mannosyltransferase 2
	GLI2	GLI family zinc finger 2
	ZNF335	Zinc finger protein 335
Epilepsy syndrome	CDKL5	Cyclin−dependent kinase−like 5
	CSTB	Cystatin B (stefin B)
	TSC1	Tuberous sclerosis 1
	CACNB4	Calcium channel, voltage−dependent, beta 4 subunit
	CLCN2	Chloride channel, voltage−sensitive 2
	PCDH19	Protocadherin 19
	KCNQ2	Potassium voltage−gated channel, KQT−like subfamily, member 2
	CLN5	Ceroid−lipofuscinosis, neuronal 5
	ATP1A2	ATPase, Na +/K + transporting, alpha 2 polypeptide
	LGI1	Leucine−rich, glioma inactivated 1
	SCN1B	Sodium channel, voltage−gated, type I, beta subunit
	ALDH5A1	Aldehyde dehydrogenase 5 family, member A1
	EFHC1	EF−hand domain (C−terminal) containing 1
Encephalitis	DDX58	DEAD (Asp−Glu−Ala−Asp) box polypeptide 58
	RPSA	Ribosomal protein SA
	TREX1	Three prime repair exonuclease 1
Demyelinating disease	PLP1	Proteolipid protein 1
	RTN4	Reticulon 4
	GALC	Galactosylceramidase
GBS	FAS	Fas (TNF receptor superfamily, member 6)

### GO network mapping reveals connections between hNPC ZIKV infection and the immune system

**Fig. 3:**
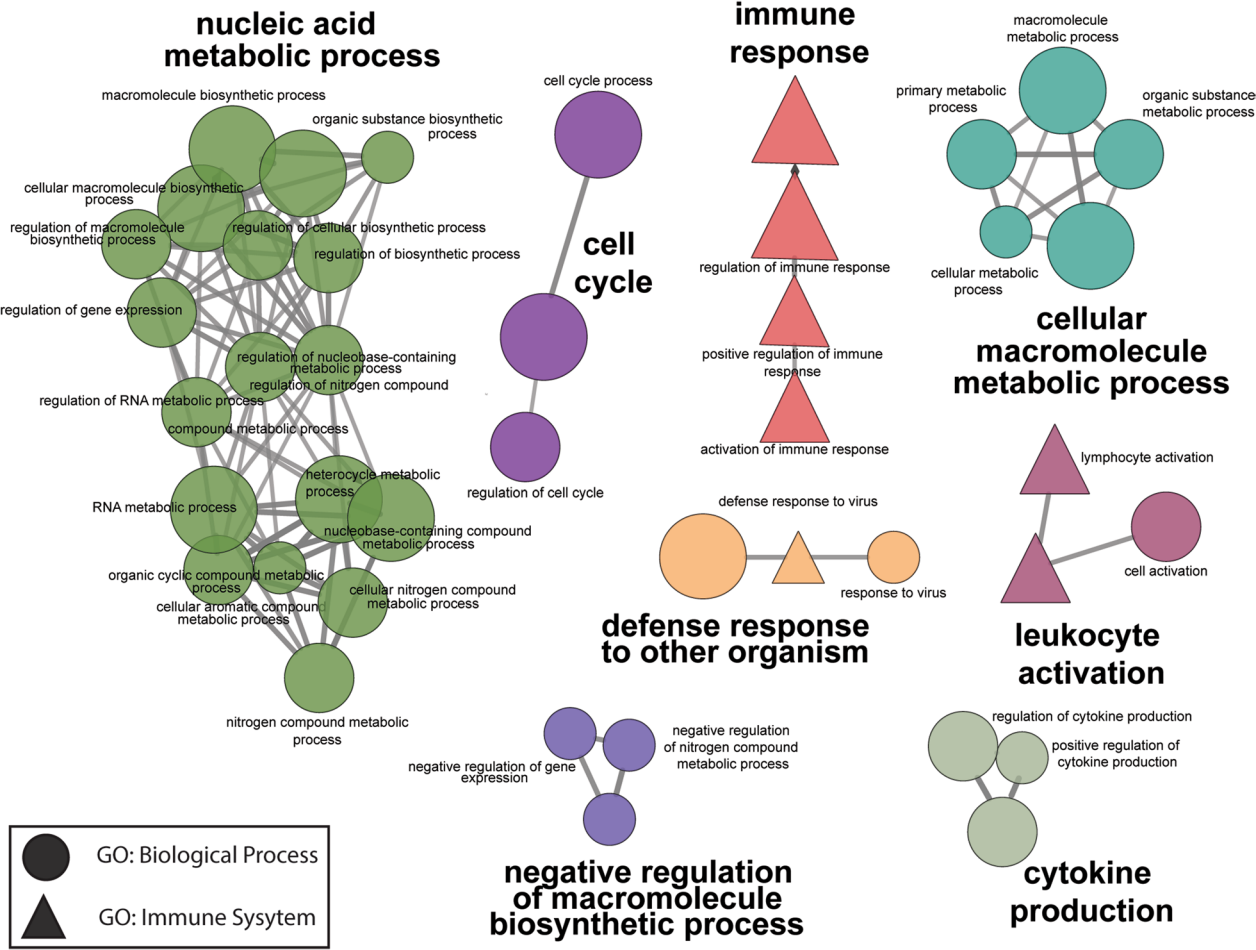
Gene ontology functional network of biological process and immune system terms. All significantly altered genes greater than 0.5 fold-change were imported into Cytoscape and Gene Ontology clustering was performed with the ClueGO plugin. The human genome was used as the background with the GO biological process and immune system terms queried for enrichment. Terms were generated such that GO Term Fusion was implemented on pathways with a less than 0.01 q-value. Groupings with less than 3 connections were excluded from the final list of networks. Terms related to cytokine and chemokine production as well as general pathogen response formed networks independent of nucleic acid and macromolecule metabolic processes

A wide variety of cell types outside the traditional immune system can respond to the threat of pathogens [19]. Thus, investigations were conducted to determine if infected hNPCs presented a pattern of gene expression consistent with a defense response. To visualize the intracellular networks regulating expression changes, a GO network map was generated (Fig. 3). All significant ZIKV DEGs greater than 0.5 absolute log fold-change were loaded into Cytoscape [20], and gene ontology clustering was performed with the ClueGO plugin [21]. The human genome was used as the background with GO biological process and immune system terms queried for enrichment. GO terms for biological processes are denoted with a circle, while GO immune system terms are denoted with a triangle. As expected, nucleic acid metabolic processes were heavily enriched and connected, representing the majority of the network and indicating that they are not a directly connected to the immune system terms. Of the eight main networks identified, four were associated with immune system response. The identified networks were immune response, cytokine production, leukocyte activation, and defense response to other organisms. Additionally, cell cycle, negative regulation of macromolecule biosynthetic process, and cellular macromolecule metabolic process terms were represented.

### Gene expression in ZIKV-infected hNPCs differs from the expression profile of CMV-infected hNPCs

A number of common human viruses are known to infect fetal NPCs, such as Herpes simplex virus 1 (HSV-1), CMV, human immunodeficiency virus (HIV), and Varicella-zoster virus (VZV), and are linked to the development of severe birth defects [6, 9, 11, 13, 22–24]. To explore if the alterations in immune related gene expression seen in ZIKV-infected hNPCs was consistent with other viral infections, we compared the ZIKV expression profile to that of CMV-infected human induced pluripotent stem (iPS) derived NPCs. This gene set provided well matched data for comparison, since the hNPCs in both studies were derived from iPS cells and implement Illumina transcript analysis [9, 23]. Additionally, hNPCs have demonstrated permissiveness to both viruses and infection in early fetal development with either virus has been linked to similar congenital neurological defects [9, 11, 23, 24].

**Fig. 4:**
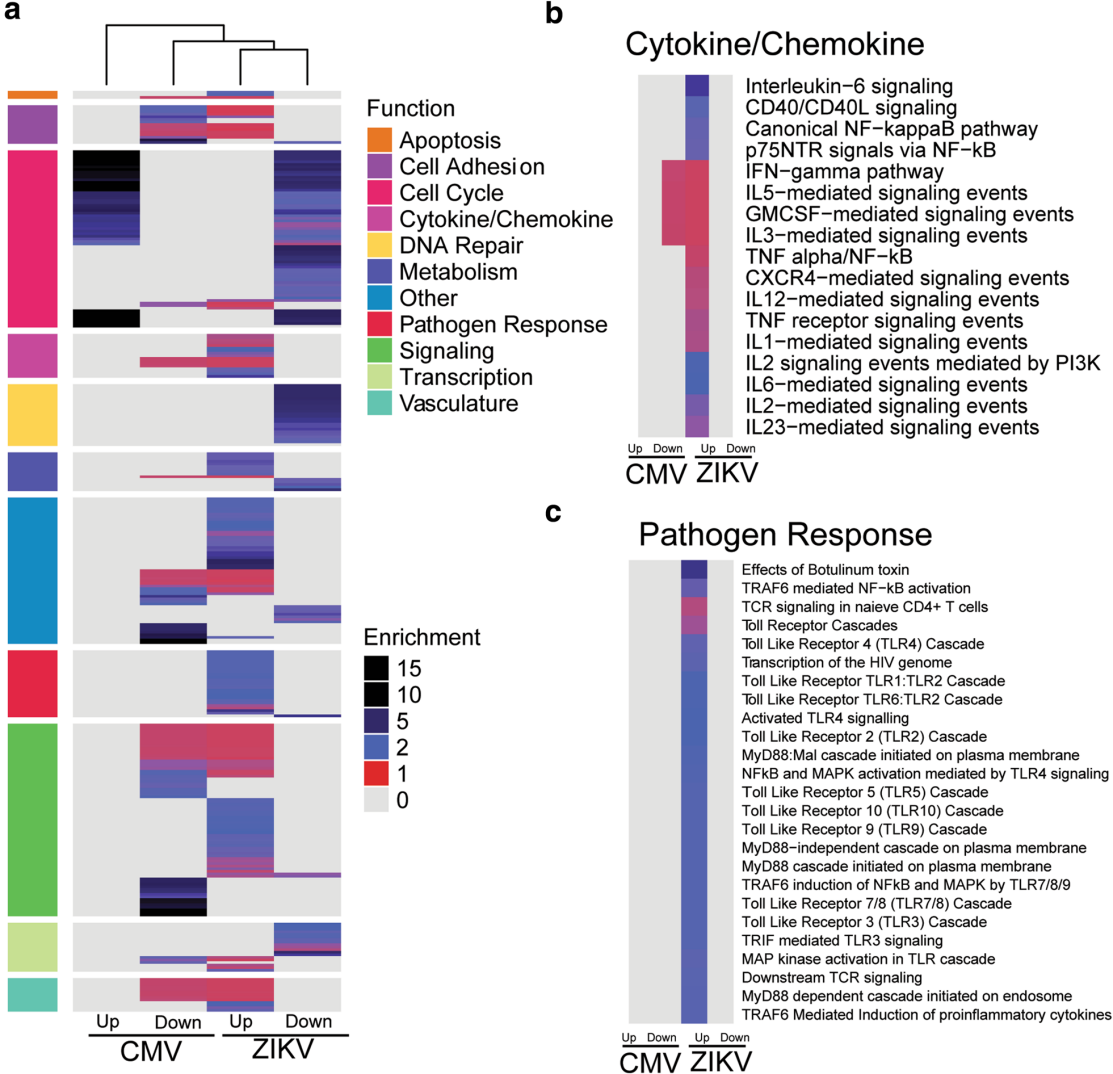
Divergent gene expression patterns in CMV and ZIKV-infected hNPCs. Genes significantly different between mock and cells infected with either CMV or ZIKV were processed identically and resulting significant biological pathways were mapped. Clustering was performed on the resulting pathways such that similar functions were grouped for clarity. The Cytokine/Chemokine and Pathogen Response clusters were enlarged to demonstrate the divergent pathway enrichment between ZIKV and CMV infection

The significant CMV related DEGs were analyzed in the same manner as the ZIKV related DEGs, such that the significant biological pathways are reported as fold enrichment. Clustering the pathways enriched by either CMV or ZIKV infection revealed apparent differences in the functional pathway enrichment patterns (Fig. 4). Forced clustering by functional pathway was used with hierarchical clustering within the groups implemented by rows. When hierarchical clustering is applied to columns, ZIKV up- and down-regulated gene pathways showed stronger similarity to each other than to CMV altered pathways (Fig. 4a). Examination of the cytokine/chemokine specific pathways likewise indicated divergent expression profiles between viruses (Fig. 4b). Several cytokine-regulated pathways were activated in ZIKV-infected NPCs, while CMV-infected hNPCs showed limited representation of these pathways. Interleukin-5 (IL-5), granulocyte-macrophage colony-stimulating factor (GM-CSF), and interleukin-3 (IL-3) mediated signaling were comparably enriched but associated with the down regulated genes in CMV but with the up regulated genes in the CMV set. Additionally, the pathogen response pathways highly represented in the ZIKV altered genes are not significantly enriched in either the CMV up or down regulated gene sets (Fig. 4c). While both viruses can infect hNPCs and cause comparable birth defects, the apparent mechanism by which these birth defects are caused appears to be unrelated between viruses.

### Immune and inflammatory responses in ZIKV-infected hNPCs

**Fig. 5:**
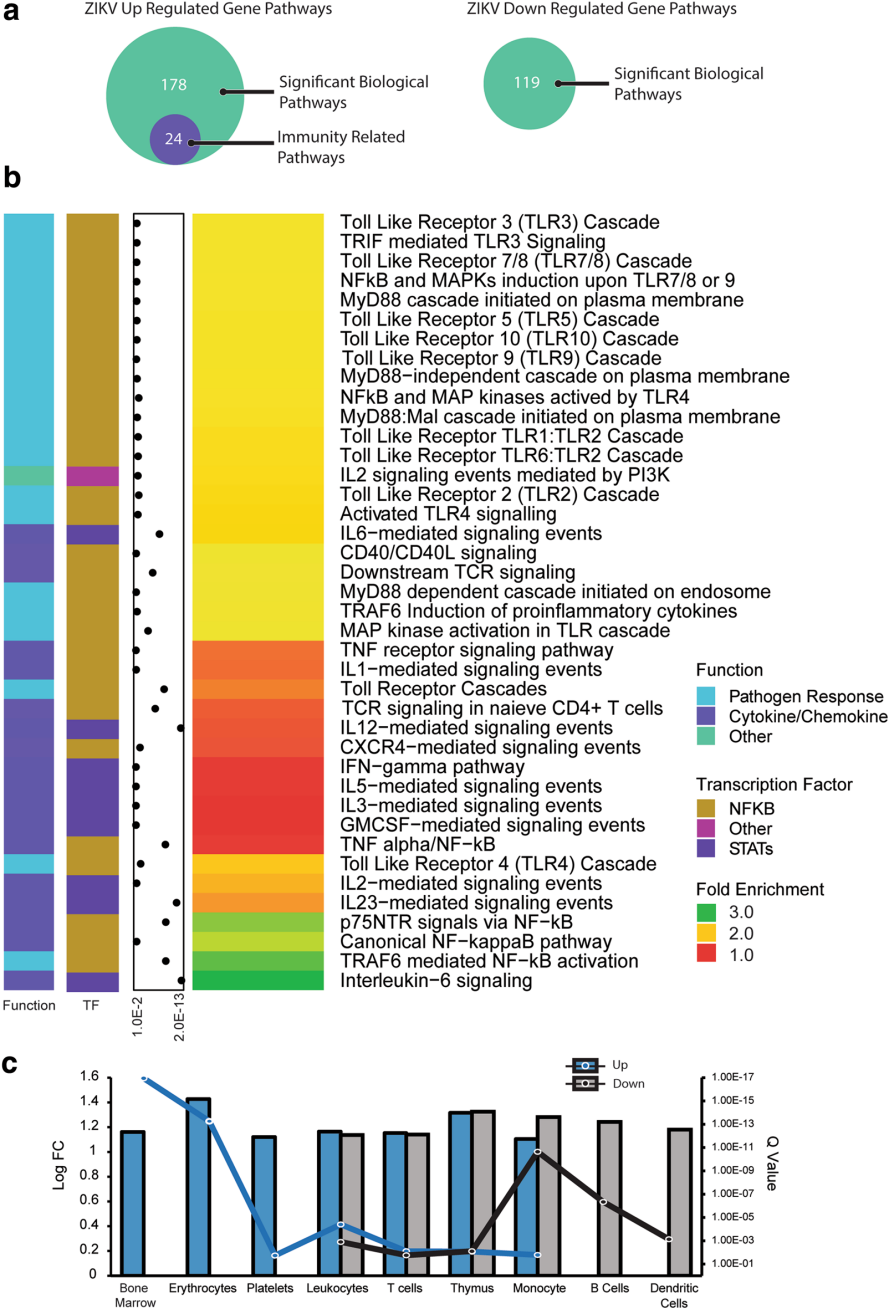
Immune specific biological pathways enriched in up-regulated genes. **a** The biological pathways associated with ZIKV transcript changes were filtered to include top immune specific pathways. **b** Using a FDR q-value <0.05, several top immune function pathways were enriched for the up-regulated gene set, although none of the pathways were enriched for significant down-regulated transcripts. The q-values for the pathway enrichment are annotated along the *left panel* as the *dot plot*. **c** Using tissue specific expression databases, up- and down-regulated genes were probed for immune related cells and tissues. Innate and adaptive immune system components were represented in both lists. (*q* < *0.05*)

Given that the immune response gene enrichment seen in ZIKV-infected hNPCs differed from CMV infection, we further investigated the potential inflammatory pathways involved. Inspection of the biological pathways significantly enriched for ZIKV DEGs revealed a pattern of immunological term enrichment in genes up-regulated by the virus. Using search terms classifying immune related pathways our query returned 24 immune related pathways out of the 178 total in the ZIKV up-regulated genes (Fig. 5a). Of the 119 significantly enriched pathways of genes down-regulated by ZIKV infection, none were related to immune function (Fig. 5a). Among the inflammatory pathways identified from the up-regulated genes, were the endosomal viral pathogen recognizing Toll-like receptor (TLR) 9 and TLR7/8 pathways which recognize CpG containing DNA and ssRNA, respectively [25]. TLR receptor signaling is primarily mediated through the adaptor protein myeloid differentiation primary response gene 88 (MyD88) in response to infection [26]. Other TLRs are located on the plasma membrane and recognize a wide variety of pathogen associated molecules [25] and were thus classified as pathogen response pathways. Additionally, several canonical inflammatory pathways were activated, including tumor necrosis factor (TNF) receptor pathways, interferon gamma (IFN-γ) signaling pathway, pathways involved in interleukin (IL)-1, IL-2, IL-3, IL-5, IL-6, IL-12, IL-23, C-X-C motif chemokine 12 (CXCL12)/CXCR4, pathway and GM-CSF mediated pathway (Fig. 5b).

Cell and tissue type analysis of the whole ZIKV DEG gene set revealed representation of tissue types associated with immune and hematological function (Fig. 5c). Tissue and cell types exclusively associated with ZIKV up-regulated genes included the bone marrow, erythrocytes, and platelets. B-cells and dendritic cells were the only cell types exclusive to the down-regulated gene set. Both up- and down-regulated genes were enriched for monocytes, T-cells, leukocytes, and the thymus. The immune specific enrichment of the genes differentially expressed in ZIKV-infected hNPCs indicate involvement of both the innate and adaptive immune system. Dendritic cells are important antigen-presenting cells (APC) while T- and B-cells are primarily responsible for adaptive response. This enrichment points to a role for ZIKV inducing NPCs to take on a yet unclear immune-modulating phenotype.

### Analysis of cytokine/chemokine associated intracellular signaling pathways

**Fig. 6:**
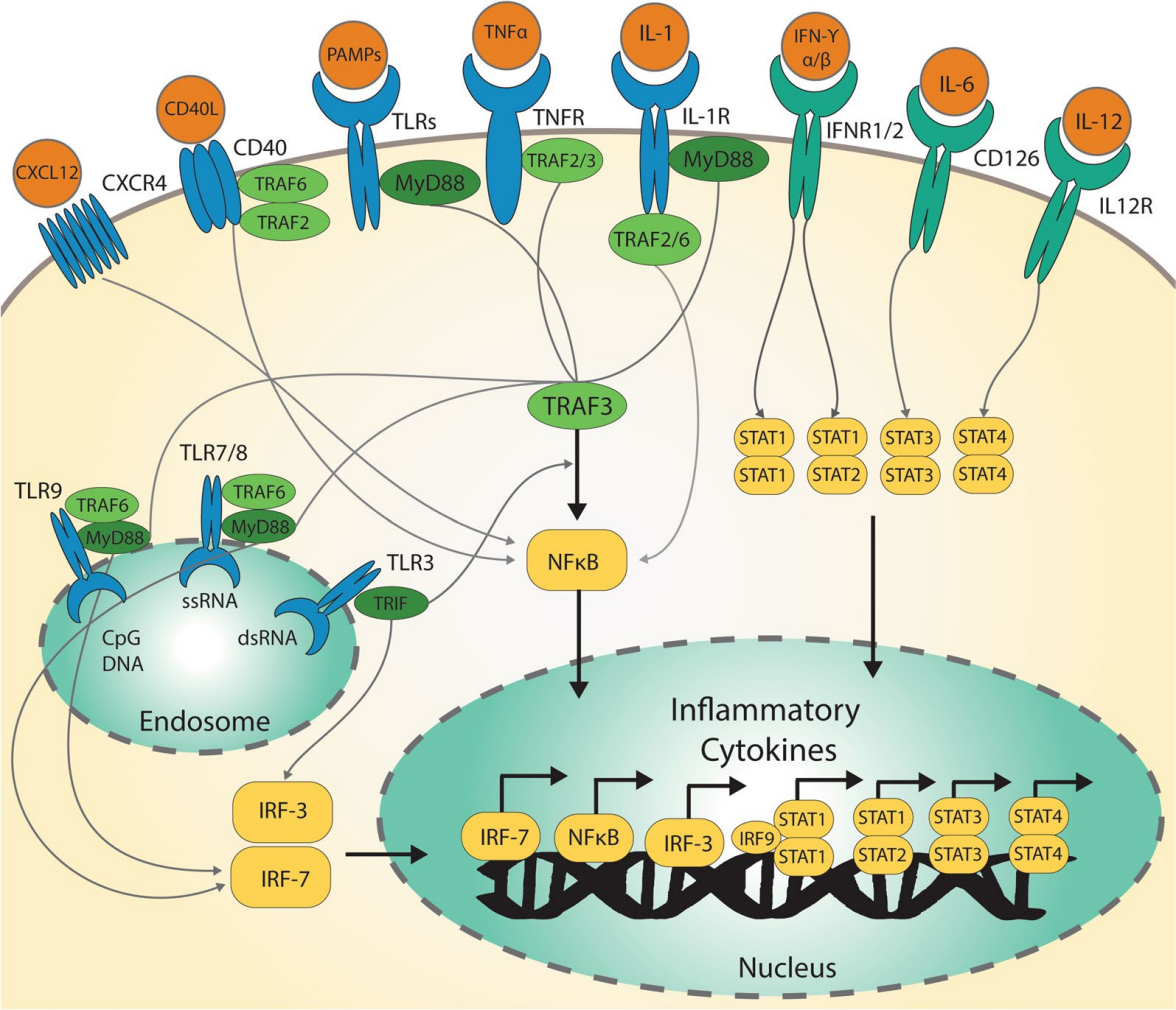
Activation of NF-κB and STATs signaling pathway in ZIKV-infected hNPCs. Cytokines (IL-1 and TNF-α), PAMPs, CD40 and CXCL12 interact with their receptors resulting in activation of the NF-κB pathway. IFN-γ, IL-6 and IL-12 binding to their receptors induces STAT dimerization and translocation to the nucleus. Activation of both the canonical NF-κB and STAT signaling pathways, results in the production of numerous inflammatory cytokines.

The two major immune cell pathways that control the production of inflammatory cytokines can be classified as being nuclear factor kappa-light-chain-enhancer of activated B cells (NF-κB) and signal transducer and activator of transcription (STAT) protein family dependent. Both NF-κB and STAT family-members are transcription factors that induce gene expression upon stimulation by wide variety of ligands. Figure 6 illustrates simplified versions of these pathways activated in ZIKV-infected NPCs. The pathogens (represented as pathogen-associated molecular patterns, PAMP) and TLR 7/8/9 pathways enriched in the ZIKV up-regulated genes (Fig. 4), induce gene expression primarily through nuclear translocation of NF-κB. Other NF-κB mediated pathways enriched in ZIKV up-regulated genes were C-X-C chemokine receptor type 4 (CXCR4), CD40, TNF, and IL-1 signaling (Fig. 4). STAT family induced genes can also result in inflammatory cytokine production upon activation of plasma membrane receptors. The enriched STAT family related signaling pathways were IFN-γ, IL-6, and IL-12 in the ZIKV up-regulated genes (Fig. 4).

## Discussion

Our results show that collectively, ZIKV induced transcriptomic alterations are associated with established clinical pathologies. Following viral infection there is a reallocation of host nucleic acids and other metabolites towards viral specific replication processes [12], thus the enrichment of biological processes related to nucleic acid metabolism is an expected finding, as well as the enrichment of cell cycle regulation. The cell cycle perturbations of infected cells suggest the initiation of a G_1_/S checkpoint and impaired progression through G_1_ [9]. A possible explanation for these cell cycle abnormalities could be inhibition of S-phase progression due to limited nucleic acid reserves as the virus reallocates them for its own replication. Down-regulation of critical epigenetic markers represents a possible driver for abnormal gene expression in infected cells. If NPCs in the developing human brain are diverted from normal development, it can result in conditions like microcephaly. Moreover, this raises questions about the possible long-term neurological effects in adult following ZIKV infection of adult neural progenitors. Although, the ZIKV associated clinical phenotypes were highly enriched for congenital CNS disorders, comparatively few genes were associated with adult CNS inflammation and demyelination diseases. This may be the due to the fact that the current study implemented the MR766 Ugandan isolate which has not been associated with an increased incidence of GBS and myelitis. As a result, future studies that employ more neoteric strains will likely observe increased representation of related genes.

The significant enrichment of canonical immune system pathways in ZIKV-infected hNPCs proved to be another unexpected finding. Our study provides evidence at the transcriptional level that two major mediators of inflammation, NF-κB and the STAT family of proteins, regulate inflammatory response in hNPCs. NF-κB is one of the main transcriptional factors activated in response to pro-inflammatory cytokines, such as TNF-α and IL-1. It is also triggered by TLRs via pathogen-associated molecular patterns (PAMPs). The Janus Kinase (JAK)-Signal transducer and activator of transcription (STAT) pathway is also a major signaling pathway that regulates inflammation and mediates the responses of target cells to inflammatory cytokines. Activation of these pathways has a central role in inflammation through the regulation of genes encoding pro-inflammatory cytokines, chemokines, and inducible enzymes such as cyclooxygenase-2 (COX2) and inducible nitric oxide synthase (iNOS) [27]. Moreover, it has recently been reported that ZIKV-infected fetal brains display a number of inflammatory markers, including diffuse astrogliosis, and activations of macrophages and microglia [9]. It is conceivable that ZIKV-infected NPCs may exert immunomodulatory and inflammatory effects on CNS cells such as neurons as well as astrocytes in an autocrine, paracrine, and juxtacrine manner, thus amplifying brain inflammation. Interestingly, although congenital CMV infection can also result in microcephaly and general impaired brain development [24], the inflammatory signals were not observed in CMV-infected hNPCs. The significance of this is still under investigation.

With the currently limited molecular data regarding transcriptomic alterations in infected host cells, large scale analysis is difficult. In spite of this, we present analysis of the currently available data in an effort to guide future studies. The RNA-seq was performed on only one infected cell type, hNPCs, thus we implemented stringent Benjamini Hochberg (BH) [28] false discovery rate (FDR) q-value cut-offs to limit false positives in enriched pathways. The use of the older MR766 isolate for the experiments likewise limits extrapolation of the results, even though it shares 88.9 % nucleotide and 96.5 % amino acid identity with the current strain [29]. Considering that reported pathogenicity has been increasing over time, the presented data likely represents a milder host response should any host transcript differences exist.

## Conclusion

In conclusion, we report that ZIKV infection induces alterations to NPC immune response gene expression. While the primary infection typically occurs through the bite of an infected mosquito, the mechanism for entry into the restricted CNS is currently unclear. Presumably the specific physiology of the fetal blood brain barrier is permissible to ZIKV as it is to other viruses [24]. Regardless, it has been shown that the virus can infect NPCs with high efficiency leading to increased apoptosis [9]. In addition, the presented study is the first to report enrichment of numerous pro-inflammatory pathways in ZIKV-infected hNPCs, indicating that hNPCs are capable of activating pro-inflammatory immune system pathways following viral infections. This represents, in addition to direct activation of apoptotic pathways, a mechanism by which the virus could induce cell death via the propagation a cytotoxic pro-inflammatory environment in the CNS. Although it is possible that ZIKV can infect immune cells in a similar manner as the related Dengue virus [30–33], the ability of ZIKV to infect hNPCs may exacerbate CNS inflammation. Consequently, a better understanding of the cellular and molecular mechanisms regulation of the cross-talk between NPCs, CNS resident cells, and immune cells may be crucial for the development of effective strategies to treat ZIKV infection.

## Methods

### Gene sets and expression analysis

We analyzed the transcriptomic sequencing (RNA-seq) data (GEO: GSE78711) of ZIKV (MR766) infected human neural progenitor cells (hNPCs). Briefly, Tang et al. infected hNPCs derived from human induced pluripotent stem (iPS) cells with ZIKV. The infected hNPCs were incubated for 56 h, a time frame already proven sufficient for a greater than 60 % infection rate. RNA extracted from ZIKV-infected samples and mock infected samples was used for library generation and sequencing [9]. For comparison to the ZIKV-infected hNPCs, the expression data from iPCS derived NPCs infected with human CMV (Ad169) was implemented (GEO: GSE35295). The cells were infected with CMV, and the RNA was extracted 24 after infection for analysis on an Illumina HumanHT-12 V4.0 expression beadchip. Three biological replicates for control and infected cells were used, and a list of significantly altered genes were generated with a BH FDR q-value cut-off of < 0.05 [23].

### Availability of data and materials

The datasets supporting the conclusions of this article are available in the gene expression omnibus (GEO) repository, http://www.ncbi.nlm.nih.gov/geo/.

### Biological process and cellular compartment analysis

Using FunRich [34], up- and down-regulated genes with associated Log_2_ FCs were analyzed for enrichment against the human annotated genome. Biological process, cellular compartment, and biological pathway significance was defined as a FDR q-value less than 0.05. All significant biological processes were presented. All 11 significant cellular compartments for the ZIKV up-regulated genes were presented, along with the top 11 most significant pathways enriched for ZIKV down-regulated genes.

### Subsetting for immune specific biological pathway enrichment

The significant biological pathways associated with both ZIKV up- and down-regulated genes were filtered such that non-immune specific biological pathways were excluded. Significance was defined as a FDR q-value less than 0.05.

### ZIKV genes associated with clinical phenotypes

Clinically relevant diseases associated with ZIKV DEGs were queried using the MalaCards [17] integrated database of human diseases. The absolute percentage of ZIKV DEGs present in the reference list derived from MalaCards was reported, as well as the total number of genes in the reference list.

### Gene ontology network analysis

DEGs with an absolute fold change greater than 0.5 were imported to Cytoscape [20], where Gene Ontology clustering was performed using the ClueGO plugin [21]. The human genome was used as the background, and the GO biological process and GO immune system terms were queried for enrichment. Terms were generated such that GO Term Fusion was implemented for pathways with a less than 0.01 FDR q-value. Groups with less than three connections were excluded from the final network.

### R packages

Heatmaps generated with the Bioconductor package ComplexHeatmap [35] within the R statistical computing environment [36].

## Abbreviations

ZIKV: Zika virus
iPSC: induced pluripotent stem cell
hNPC: human neural progenitor cell
CMV: cytomegalovirus
GBS: Guillain–Barré syndrome
GO: gene ontology
FC: fold-change
MOZ/MORF: monocytic leukemia zinc-finger protein/related factor
DEG: differentially expressed genes
CNS: central nervous system
HSV-1: herpes simplex virus 1
HIV: human immunodeficiency virus
VZV: varicella-zoster virus
GM-CSF: granulocyte–macrophage colony stimulating factor
IL: interleukin
TLR: toll-like receptor
MyD88: myeloid differentiation primary response gene 88
TNF: tumor necrosis factor
IFN: interferon
CXC: C-X-C motif chemokine
APC: antigen-presenting cells
STAT: signal transducer and activator of transcription
NF-κB: nuclear factor kappa-light-chain-enhancer of activated B cells
PAMP: pathogen-associated molecular patterns
JAK: Janus kinase
COX2: cyclooxygenase-2
iNOS: inducible nitric oxide synthase

## References

[1] Faye O, Freire CC, Iamarino A, Faye O, de Oliveira JV, Diallo M (2014). Molecular evolution of Zika virus during its emergence in the 20(th) century. PLoS Negl Trop Dis.

[2] Lazear HM, Diamond MS (2016). Zika virus: new clinical syndromes and its emergence in the western hemisphere. J Virol.

[3] Cao-Lormeau V-M, Blake A, Mons S, Lastère S, Roche C, Vanhomwegen J (2016). Guillain-Barre syndrome outbreak associated with Zika virus infection in French Polynesia: a case-control study. Lancet.

[4] Zammarchi L, Tappe D, Fortuna C, Remoli ME, Gunther S, Venturi G, et al. Zika virus infection in a traveller returning to Europe from Brazil, March 2015. Euro Surveill. 2015;20(23). 10.2807/1560-7917.es2015.20.23.21153 26084316

[5] Olson JG, Ksiazek TG, Suhandiman, Triwibowo (1981). Zika virus, a cause of fever in Central Java, Indonesia. Trans R Soc Trop Med Hyg.

[6] Ventura CV, Maia M, Bravo-Filho V, Góis AL, Belfort R (2016). Zika virus in Brazil and macular atrophy in a child with microcephaly. Lancet.

[7] Mlakar J, Korva M, Tul N, Popović M, Poljšak-Prijatelj M, Mraz J (2016). Zika virus associated with microcephaly. N Engl J Med.

[8] Kleber de Oliveira W, Cortez-Escalante J, De Oliveira WT, do Carmo GM, Henriques CM, Coelho GE (2016). Increase in reported prevalence of microcephaly in infants born to women living in areas with confirmed Zika virus transmission during the first trimester of pregnancy—Brazil, 2015. MMWR Morb Mortal Wkly Rep.

[9] Tang H, Hammack C, Ogden Sarah C, Wen Z, Qian X, Li Y (2016). Zika virus infects human cortical neural progenitors and attenuates their growth. Cell Stem Cell.

[10] Dollard SC, Grosse SD, Ross DS (2007). New estimates of the prevalence of neurological and sensory sequelae and mortality associated with congenital cytomegalovirus infection. Rev Med Virol.

[11] Luo MH, Hannemann H, Kulkarni AS, Schwartz PH, O’Dowd JM, Fortunato EA (2010). Human cytomegalovirus infection causes premature and abnormal differentiation of human neural progenitor cells. J Virol.

[12] Martin EM, Malec J, Sved S, Work TS (1961). Studies on protein and nucleic acid metabolism in virus-infected mammalian cells. 1. Encephalomyocarditis virus in Krebs II mouse-ascites-tumour cells. Biochem J.

[13] Garcez PP, Loiola EC, Madeiro da Costa R, Higa LM, Trindade P, Delvecchio R (2016). Zika virus impairs growth in human neurospheres and brain organoids. Science.

[14] Mahmood S, Ahmad W, Hassan MJ (2011). Autosomal recessive primary microcephaly (MCPH): clinical manifestations, genetic heterogeneity and mutation continuum. Orphanet J Rare Dis.

[15] Bond J, Roberts E, Springell K, Lizarraga SB, Scott S, Higgins J (2005). A centrosomal mechanism involving CDK5RAP2 and CENPJ controls brain size. Nat Genet.

[16] Yang XJ, Ullah M (2007). MOZ and MORF, two large MYSTic HATs in normal and cancer stem cells. Oncogene.

[17] Rappaport N, Nativ N, Stelzer G, Twik M, Guan-Golan Y, Iny Stein T, et al. MalaCards: an integrated compendium for diseases and their annotation. Database (Oxford). 2013;bat018. 10.1093/database/bat018 PMC3625956 23584832

[18] Aggarwal A, Mittal H, Patil R, Debnath S, Rai A (2013). Clinical profile of children with developmental delay and microcephaly. J Neurosci Rural Pract.

[19] Anders HJ, Banas B, Schlondorff D (2004). Signaling danger: toll-like receptors and their potential roles in kidney disease. J Am Soc Nephrol.

[20] Shannon P, Markiel A, Ozier O, Baliga NS, Wang JT, Ramage D (2003). Cytoscape: a software environment for integrated models of biomolecular interaction networks. Genome Res.

[21] Bindea G, Mlecnik B, Hackl H, Charoentong P, Tosolini M, Kirilovsky A (2009). ClueGO: a cytoscape plug-into decipher functionally grouped gene ontology and pathway annotation networks. Bioinformatics.

[22] Nowakowski TJ, Pollen AA, Di Lullo E, Sandoval-Espinosa C, Bershteyn M, Kriegstein AR (2016). Expression analysis highlights AXL as a candidate Zika virus entry receptor in neural stem cells. Cell Stem Cell.

[23] D’Aiuto L, Di Maio R, Heath B, Raimondi G, Milosevic J, Watson AM (2012). Human induced pluripotent stem cell-derived models to investigate human cytomegalovirus infection in neural cells. PLoS One.

[24] Cheeran MCJ, Lokensgard JR, Schleiss MR (2009). Neuropathogenesis of congenital cytomegalovirus infection: disease mechanisms and prospects for intervention. Clin Microbiol Rev.

[25] Takeda K, Akira S (2004). TLR signaling pathways. Curr Protoc Immunol.

[26] Akira S, Takeda K (2004). Toll-like receptor signalling. Nat Rev Immunol.

[27] Baud V, Karin M (2009). Is NF-[kappa]B a good target for cancer therapy? hopes and pitfalls. Nat Rev Drug Discov.

[28] Benjamini Y, Hochberg Y (1995). Controlling the false discovery rate: a practical and powerful approach to multiple testing. J R Stat Soc Series B Stat Methodol.

[29] Lanciotti RS, Kosoy OL, Laven JJ, Velez JO, Lambert AJ, Johnson AJ (2008). Genetic and serologic properties of Zika virus associated with an epidemic, Yap State, Micronesia, 2007. Emerg Infect Dis.

[30] Simmons CP, Farrar JJ, van Vinh Chau N, Wills B (2012). Dengue. N Engl J Med.

[31] Nakao S, Lai CJ, Young NS (1989). Dengue virus, a flavivirus, propagates in human bone marrow progenitors and hematopoietic cell lines. Blood.

[32] Kelley JF, Kaufusi PH, Volper EM, Nerurkar VR (2011). Maturation of dengue virus nonstructural protein 4B in monocytes enhances production of dengue hemorrhagic fever-associated chemokines and cytokines. Virology.

[33] La Russa VF, Innis BL (1995). Mechanisms of dengue virus-induced bone marrow suppression. Baillieres Clin Haematol.

[34] Pathan M, Keerthikumar S, Ang CS, Gangoda L, Quek CY, Williamson NA (2015). FunRich: an open access standalone functional enrichment and interaction network analysis tool. Proteomics.

[35] Zuguang G. ComplexHeatmap: making complex heatmaps. R package version 1.6.0 ed2015.

[36] R Core Team (2015). R: a language and environment for statistical computing.

